# A role for spinal cord hypoxia in neurodegeneration

**DOI:** 10.1038/s41419-019-2104-1

**Published:** 2019-11-13

**Authors:** Elena Hernandez-Gerez, Ian N. Fleming, Simon H. Parson

**Affiliations:** Institute of Medical Sciences University of Aberdeen Foresterhill Aberdeen, AB25 2ZD Scotland, UK

**Keywords:** Neurodegeneration, Spinal cord diseases

## Abstract

The vascular system of the spinal cord is particularly complex and vulnerable. Damage to the main vessels or alterations to the regulation of blood flow will result in a reduction or temporary cessation of blood supply. The resulting tissue hypoxia may be brief: acute, or long lasting: chronic. Damage to the vascular system of the spinal cord will develop after a traumatic event or as a result of pathology. Traumatic events such as road traffic accidents, serious falls and surgical procedures, including aortic cross-clamping, will lead to an immediate cessation of perfusion, the result of which may not be evident for several days, but may have long-term consequences including neurodegeneration. Pathological events such as arterial sclerosis, venous occlusion and spinal cord compression will result in a progressive reduction of blood flow, leading to chronic hypoxia. While in some situations the initial pathology is exclusively vascular, recent research in neurodegenerative disease has drawn attention to concomitant vascular anomalies in disorders, including amyotrophic lateral sclerosis, spinal muscular atrophy and muscular sclerosis. Understanding the role of, and tissue response to, chronic hypoxia is particularly important in these cases, where inherent neural damage exacerbates the vulnerability of the nervous system to stressors including hypoxia.

## Facts

- Damage to the spinal cord vascular system results in acute or chronic hypoxia.
- Hypoxia causes progressive and irreversible damage, which can be initially difficult to detect.
- Hypoxia is damaging to neurones, especially those already affected by intrinsic, neurological disease.

## Open questions

- What is the effect of chronic hypoxia on the nervous system?
- Is undiagnosed chronic hypoxia a causative or confounding factor in neurodegenerative disease?
- Should the functionality of the spinal cord vascular system be taken into account in the treatment of neurodegenerative disease?

## Background

Neurodegenerative diseases encompass a wide range of disorders that have the deterioration and death of neurones in the central nervous system (CNS) as a common link. While often grouped together, the origin of the pathology is highly variable, and may be intrinsic, due to genetic factors, extrinsic, caused by external damage or a combination of risk factors. Even within the same disease, in cases like amyotrophic lateral sclerosis (ALS), there is a broad array of mutated genes, such as SOD-1 or SEXT, which result in the same symptomatology by which they are then grouped^1,2^. While neurone-specific alterations tend to be very disease specific, and research has shown that frequently there is some sort of peripheral pathology that exacerbates neural damage. For example, inflammation in ALS and multiple sclerosis, or microglial damage in brain pathologies such as Alzheimer’s, aggravates the underlying disease^3,4^. Recently, there has been significant research into vascular damage and the role of hypoxia in brain neurodegeneration in diseases such as Alzheimer’s and other forms of dementia. This line of research has not been explored in depth in relation to spinal cord neurodegeneration. However, the existing studies show great potential to provide further understanding of disease aetiology and risk factors, especially considering that they are relevant to inherited disease and traumatic injury of the spinal cord.

## Spinal cord vascular system

**Fig. 1:**
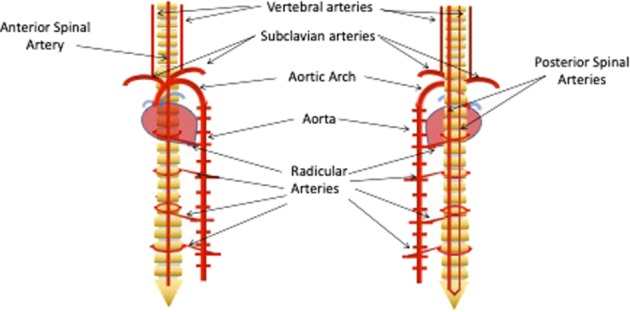
The blood supply of the spinal cord originates mainly from the aorta. The network of vessels that surround the cord is connected by three main vessels: the anterior spinal artery and the two posterior spinal arteries

Any research into the role of hypoxia in neurodegeneration in the spinal cord needs to consider whether the hypoxia is acute or chronic (Table 1). Acute hypoxia is short term, caused by a transient decrease in blood flow to an area, followed by a critical decrease in oxygen level. In contrast, chronic hypoxia results from a long-term reduction of normal oxygen levels^5^. Hypoxia studies also need to take into account the complexity of the vascular supply, and most importantly its particularly low efficiency^6^. The main vessel network develops during the first 6 months of embryonic development, and then its layout remains relatively unchanged to adulthood^7^. Extraspinal vessels (chiefly branches of the aorta^8^) are responsible for the majority of the blood flow that arrives into the system^9^. They connect with the intrinsic arterial system, which can be divided into a central and a peripheral system. The central system supplies mainly the grey matter through the sulcal arteries, which are longitudinally connected by the anterior spinal artery^10^. Depending on the type of radicular artery, they can also feed the dura mater or the nerve roots close to them, and sometimes they can be feeders of the anterior or posterior spinal arteries^11^. The white matter is mostly supplied by the peripheral system or pial network, which covers the exterior of the spinal cord, from where they branch perpendicularly into the cord. Similarly to the sulcal arteries, the pial network is connected longitudinally by the two posterior spinal arteries^10^. The posterior and anterior arteries are highly interconnected along the spinal cord at a capillary level^12^, with the main point of contact being at the termination of the cauda equina^12,13^ (Fig. 1).

**Table 1:** Summary of the major causes of vascular damage to the spinal cord; the sites of initial damage and the long-term consequences of that damage

		Initial damage	Consequences	Key references
Hypoxia caused by traumatic events	Acute spinal cord hypoxia	Traumatic accidents (e.g. car accidents, falls), surgery can cut temporarily blood flow to the spinal cord	Neural necrosis within 6 h and up to 34–48 after hypoxic episode. Long-lasting damage, normally irreversible	Richards et al.^23^, Gravereaux et al.^24^, Ahuja et al.^25^, Kato et al.^26^, Long et al.^38^
	Long-term spinal cord compression	Damage to the spinal cord can result in chronic compression of the spinal cord paired with a prolonged decrease of the blood supply	Decrease of vascular microvasculature. Slow neural damage, eventually irreversible (after 9 weeks)	Cheng et al.^37^, Long et al.^38^, Kurokawa et al.^39^, Kasahara et al.^40^
Hypoxia due to chronic disease	Vascular alterations	Vascular pathology (e.g. arteriovenous fistulas) can result in a prolonged decrease of the blood supply	Similar to spinal cord compression. Shown to be damaging to oligodendrocytes (demyelination)	Hurst et al.^49^, Larsson et al.^50^, Jellemaet al.^51^, Duncombe et al.^54^, Shibata et al.^57^
	Motor neurone disease and muscular sclerosis	Vascular anomalies have been detected in some neurodegenerative diseases (e.g. ALS, SMA), resulting in alterations of the normal blood supply	Neural damage and demyelination likely to be increased. Potential negative effect in neurone-focused treatments	Somers et al.^61^, Zhong et al.^62^, Nobutoki and Ihara^63^, Miyazaki et al.^64^, Davies et al.^72^, Desai et al. n.d., Hua et al.^81^

The spinal cord is particularly vulnerable to damage within the vascular network. While tissue oxygen levels at the spinal cord are the same as the oxygen levels in the brain (35–39 mmHg), the blood flow is between 40 and 60% lower^11^. The level of vulnerability may also vary depending on the spinal cord section. The cervical area is well supplied, and receives its main blood flow coming from radicular arteries branching from the vertebral and subclavian arteries. The superior extent of the upper anterior spinal artery is formed at this point by the union of the vessels arising from each vertebral artery, while the superior extent of the posterior spinal arteries are formed by anastomoses between branches of the vertebral arteries and posterior inferior cerebral arteries. The lower cervical spinal cord is supplied by a vessel originating from the deep cervical artery, which in turn originated from the costocervical trunk that branches from the subclavian artery^14^. The sacral sections have numerous connections to the lateral sacral arteries^15^. Both the thoracic and the lumbar areas feed mainly from segmental arteries branching directly from the aorta. However, the distance between the main points of entry into the spinal cord blood network is a lot greater in the thoracic area^11^.

**Fig. 2:**
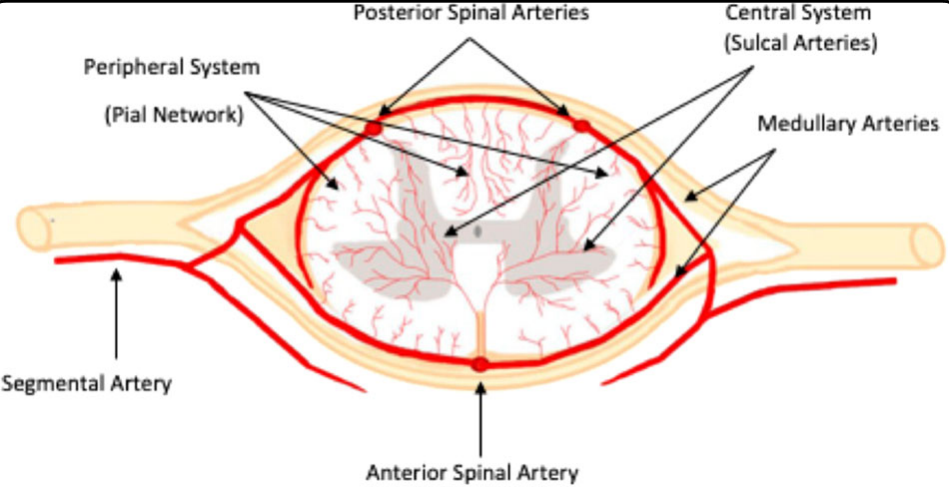
The three major blood vessels feed the medulla through the peripheral and central systems

The lower number of segmental arteries means that occlusion of any artery feeding into the thoracic spinal cord can potentially be significantly more damaging than in any other region, and that both the thoracic and lumbar areas are far more at risk in the event of aortic occlusion or damage. This is especially true of the artery of Adamkiewicz, the main radicular artery of the spinal cord. This artery branches from the aorta at a variable point in the lumbar or thoracic region, but most frequently between T9 and T12, and rather less frequently between T5 and T8 or L1 and L2. It passes though the intervertebral foramen, and makes a hairpin turn in the anterior spinal cord, where it anastomoses with the anterior spinal artery^16^. The occlusion of the artery of Adamkiewicz has been frequently shown to result in paraplegia^17^. Within the cord (Fig. 2), the capillary network density is always much higher in grey matter compared with white matter^18^. Grey matter artery density can also vary, as it is correlated to the metabolic demand of each region of the spinal cord^19^. Within grey matter, there is a decrease in vascularity at the ends of the posterior horns, their irrigation being decreased in comparison with the rest of the grey matter^11^. As motor neurones are situated in this area of lower vessel density, they are at higher risk than other cells of being affected by any alterations to the blood supply or any damage to the microvasculature. A low vessel density increases the likelihood of the collateral circulation being insufficient to protect the surrounding tissue from damage in the case of loss of perfusion, and therby contributes to the higher vulnerability of the tissue.

Haemodynamics are also highly complex, and can make the consequences of vessel damage difficult to predict. Blood flow can be ascending or descending within the cord, which creates numerous watershed areas where opposing currents meet^20^. Watershed areas have a higher likelihood of becoming hypoxic during any event that results in nearby areas being subjected to abnormal pressure^21^. Again, this can be particularly damaging at the thoracic region, where the larger distance between radicular arteries results in larger watershed areas^11^. Blood flow can be regulated rapidly by mechanisms that regulate blood and tissue pressure, even changing its direction, but this regulation may not work correctly in an unhealthy spinal cord. The fragility of this system needs to be taken into consideration in cases of non-traumatic hypoxia. Chronic near- or actual hypoxic conditions may mean that any disease-related (acute) hypoxia may not induce a normal protective response, and could potentially aggravate degeneration due to improper regulation of cellular processes.

## Acute hypoxia in spinal cord traumatic injuries

Traumatic injuries are one of the main potential causes of damage to the spinal cord. Spinal cord injuries affect thousands of people every year, and their effect can last a lifetime^22^. These injures are mainly caused by car accidents or falls, as well as violence and sport-related accidents, so they can affect a wide range of people^23^. Similarly, surgery involving vessels that supply the spinal cord, such as those clamped during surgery for thoracoabdominal aneurysms, generates a considerable risk of damage due to ischaemia resulting from the operation, with very similar consequences^24^. There are several factors in a traumatic injury that can lead to ischaemia in the spinal cord. Both the decrease/loss of perfusion and also autonomic nerve dysfunction can lead to loss of blood pressure regulation and excessive relaxation/dilation of blood vessels, generally resulting in acute hypotension. If the damage is significant and affects pulmonary function, oxygen levels in blood may also be reduced^25^. Even after a short (15–20 min) episode of hypoxia, neural necrosis will rapidly ensue within 6 h, and damaged cells will continue to die from apoptosis for 24–48 h^26^. This last apoptotic stage can be greatly aggravated by the cytokine cascades and microglia that were activated by the initial necrotic cell damage^27^. Although this damage can be long lasting, and normally irreversible, these changes due to acute hypoxia can be triggered by a very short period of initial hypoxic damage.

## Chronic hypoxia due to vessel compression

While acute compression, which significantly reduces blood flow, can have a very similar outcome to a spinal cord injury^28^, chronic compression can be more difficult to detect but may still have long-term consequences. Chronic compression has been hypothesised as the origin of several myelopathies. Cervical myelopathies appear in elderly individuals and are one of the most common spinal cord disorders at late ages^29^. They are caused by compression of the spinal cord canal, generally caused by degeneration or abnormal ossification of the ligaments and vertebrae^30^. Unlike spinal cord injury, where the main damage is related to the main vessels, the vascular hypothesis of this disease considers damage to the microvasculature a more likely cause of the ischaemia^31^. Other myelopathies in this group include Surfer’s myelopathy, caused by hyperextension of the back due to the inadequate practice of the sport^32^, or Hirayama disease, a disorder that appears as progressive weakness and dysfunction of the upper body and limbs in young people^33,34^. It is also possible for spinal cord vascular compression to appear as a secondary effect of metastatic tumours^35^. Taken together, the incidence of these diseases is low; a study of Hirayama disease in Japan found only 333 cases between 1996 and 1998^36^, and the prevalence of degenerative cervical myelopathy in the United States has been estimated at 4.04/100,000 people per year^30^. Consequently, most of the research on the effects of long-term spinal cord compression has been done in mouse models. Chronic compression has been shown to result in a decrease of vascular density^37^, especially of the microvasculature^38^, and to a significant decrease in spinal cord blood flow^39^. Damage of this kind can be very slow to develop and will eventually cause irreversible damage, but might be reversible if detected in the early stages. Studies on rats showed that neural damage could be recovered from when chronic compression was present for up to 6 weeks, but this capacity for recovery disappeared if the compression lasted longer than 9 weeks^40^. While early diagnosis for early relief of the compression would probably be the best option, for example with surgery^41^, diagnosis tends to happen after neurological symptoms have already appeared, which is by definition too late. This applies both to arterial and venous compression; while most research is focussed on the former, there is also related compression of the venous plexus at the cauda equina, which could be correlated with neurodegeneration, particularly with lumbar stenosis^42–44^.

## Chronic hypoxia due to vascular alterations

Blood flow to the spinal cord can also be altered by vascular damage. These cases, while scarce, are particularly interesting due to the damage being caused exclusively by alterations of the blood flow. Both traumatic injuries and compression damage have a physical trauma factor that can affect neuronal well-being and alter the normal state of the surrounding tissue. The disarray of the normal blood distribution can have several causes. An unusual example would be a case where a myelopathy developed due to cholesterol-related arteriosclerosis^45^. Most cases are related to arteriovenous malformations, the most common being spinal–dural arteriovenous fistulas, with a prevalence of 5–10 cases per million^46^. This disorder appears in the form of vascular lesions at the spinal cord, sometimes due to genetic diseases, and generally in the thoracic area^47,48^. The lesions tend to result in alterations of normal blood flow, arterialisation of intramedullary veins and eventual ischaemia and necrosis^49–51^. This type of disease is frequently misdiagnosed, due to the symptoms being nerve-related (extremity weakness, pain and sensory malfunction^48,52^), and therefore confused with other forms of degenerative spinal disease^48^. While there is little research specific to spinal cord in this area, lately it has become more relevant in brain disease, particularly in small-vessel disease. Small-vessel disease effects tend to be long term. It is considered one of the main causes for vascular cognitive impairment, and is related to about 45% of new dementia cases were diagnosed globally every year^53^. Models for this disease focusing on hypoperfusion of the nervous tissue have shown that a decrease in the blood flow results in chronic hypoxia, particularly in the white matter^54^. It has also been shown to cause alterations in the blood-brain barrier and inflammation^54–56^. Particularly relevant for potential spinal cord research is that chronic hypoxia has been shown to be particularly damaging for oligodendrocytes and oligodendrocyte precursors, resulting in axon demyelination^57^.

## Role of chronic hypoxia in motor neurone disease and muscular sclerosis

Alterations of the vascular system have proven to be sufficient to trigger neural pathology, suggesting that vascular alterations in diseases where neurones are already affected by other factors could be exacerbating the pathology. Two families of disease where this may be a factor are amyotrophic lateral sclerosis (ALS) and spinal muscular atrophy (SMA). Both of these diseases commonly have their origin in genetic defects, whether there are several genes involved, as in ALS^2^, or just one, as in SMA^58^. They overlap in several aspects of the pathologies^59,60^, and vascular defects could also be a common trait^61–64^. Alterations in the superoxide dismutase-1 (SOD-1) gene are correlated with 20% of familial and 2% of sporadic cases of ALS^65^, which are known to affect the vascular system, particularly the microvasculature^62^. Histological analysis has shown alterations in the structure of the neurovascular unit^64^, which importantly, occur prior to neurone damage^62,64^. This in turn suggests that they may influence the onset of neurodegeneration in ALS, especially since cell death has been linked to an increase in hypoxia biomarkers^66,67^, and exposure to intermittent hypoxia advances disease progression in mouse models^68^. Similarly, histology of SMA spinal cords has shown a significant reduction in vascular density, along with hypoxia in motor neurone cell bodies^61^. It has also been speculated that astrocytic malfunction could affect capillary blood flow regulations, which along with high levels of vasoconstrictor hormones could increase the chances of chronic hypoperfusion^63^.

Besides ALS and SMA, there are other motor neurone diseases where chronic hypoxia could be a factor. The role of chronic hypoxia in axonal demyelination is of particular interest in demyelinating disease like multiple sclerosis (MS)^69^. Unlike ALS and SMA, the causes of MS are far from clear, but are thought to be a mix of complex genetic factors and external factors like Epstein–Barr virus infecton^70,71^. Research done in MS shows that not only can hypoxia result in damage following a similar pattern to the lesions in early disease, but that an animal model would show less damage under the same conditions if supplied with highly oxygenated breathing air (80–95% oxygen)^72,73^. This shows that chronic hypoxia likely plays a role in the disease, which fits with the findings of decreased vascularity in the brain^74^, even if there is no information about spinal cord vascularity. It also opens the possibility of highly oxygenated air used as a treatment to slow down the damage caused by MS.

These findings are likely also relevant in SMA, where a novel treatment has been recently approved for patients. SMA is the result of the homozygous mutation of the SMN1 gene, resulting in a failure to produce the survival motor neurone protein (SMN)^58^, which is necessary for survival beyond the embryonic stage^75^. SMA only occurs as a disease because of the existence of the survival motor neurone 2 (SMN2) gene, which is identical to SMN1 save for a single mutation at exon 7 that results in its exclusion during splicing. Due to this variation, only a small amount of functional SMN is produced by this SMN2 gene^76^. Multiple copies of the SMN2 gene allow for a sufficient amount of SMN protein to be produced to ensure survival, but not to ensure full health. The treatment consists of intrathecal injections of an antisense oligonucleotide that promotes inclusion of exon 7 in SMN2, resulting in higher levels of full-length SMN protein being produced in patient cells. While promising, the delivery by intrathecal injections means that the vascular system remains untreated^77^. SMA has always been considered a neurodegenerative disease, but SMN is a ubiquitous protein that is expressed in every cell and tissue^78^. More recently, considerable evidence has shown a significant systemic pathology^61,79,80^ that includes organ, blood cell and vascular alterations. Given the findings of vascular dysfunction at a pre-symptomatic stage, and considering that this treatment is delivered to very young children, future damage caused by chronic dysfunction of perfusion cannot be ignored. This is supported by mouse models of acute SMA, where peripheral delivery of therapeutics has been shown to be required for long-term effectiveness^81^.

## Chronic and acute hypoxia response

**Fig. 3:**
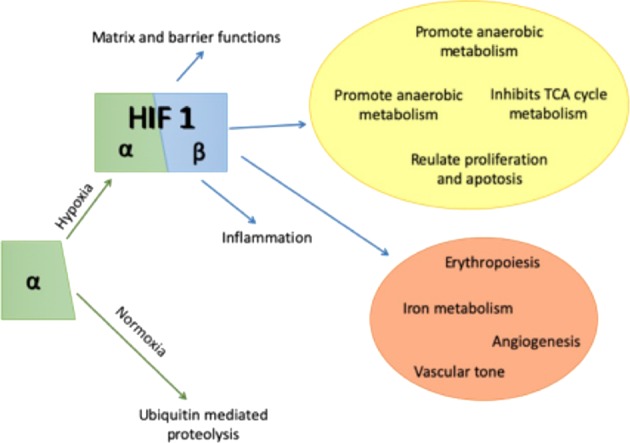
Hypoxia induces a HIF-1 mediated cellular response that involves a large range of cellular and systemic functions related to neuronal survival

In order to better predict the possible consequences of spinal cord hypoxia on neurones, it is necessary to consider the natural response of the organism to hypoxia. The basis of the hypoxia response is relatively well known. It is based on the promotor protein HIF1, whose activation depends on the α-subunit of the protein that accumulates when cellular oxygen levels fall^82^. HIF1 (Fig. 3) will trigger the production of a wide range of proteins among whose functions are regulation of apoptosis, metabolism and angiogenesis^83^, in a response time that can be as short as 30 min^84^. However, the HIF1 response tends to have a more significant role in the response to acute hypoxia. Its activation requires very low oxygen levels (around 1% O_2_), and within a relatively short period of time, <72 h, it is downregulated^85^. In chronic hypoxia, the main response appears to come from a different HIF form: HIF-2α. This protein appears during milder hypoxia (around 5% O_2_)^85^ and remains active for a much longer period of time^86^ during which its major function seems to be in promoting vascular development^87^ and increasing erythropoiesis^88^. Understanding the nature of this response is especially important in cases where the vascular system has already been shown to malfunctioning^51,62,64^. In such cases, the main response of the organism to ameliorate damage may be completely ineffective, especially in diseases like ALS, where the HIF1 response has been shown to be defective in monocytes, suggesting that the hypoxia molecular response could be affected^89^. It has been shown that dysregulation of HIF1 can result in a decrease of vascular endothelial growth factor (VEGF) expression, with a consequent decrease in angiogenesis^90,91^. Outside of disease, the main situation where an organism needs to adapt to lower-than-normal oxygen conditions is related to changes in altitude^92^. Research in this area has shown that the first response in the brain is to increase blood flow^93^, followed by an increase of red cell volume^92^ and an increase in angiogenesis^94^. Assuming that the same pattern occurs in the spinal cord, it is hard to say what could happen in diseases where vascular development and blood flood regulation are already affected.

## Conclusion

Research on neurodegeneration is frequently centred on neurone-specific responses and processes. However, it is necessary to remember that regulation of the environment where the neurones are located depends significantly on the tissues surrounding them. Vascular tissue has been a significant focus for research in brain neurodegeneration^95,96^, yet there is still little research in this area with respect to spinal cord neurodegeneration. Considering the well-established fact that poor blood distribution and ischaemia alone can cause neural damage^51^, it is likely that the vascular system in its entirety (heart, vessels and circulating cells) is a factor that must be carefully considered in any neurodegenerative disease. This is especially relevant in diseases like ALS or SMA, where neurones have already been debilitated by other factors that increase their vulnerability to hypoxic stress.
